# The Warren Prize

**Published:** 1875-11

**Authors:** 


*The Warren Prize.*

We would call attention to the advertisement of the Warren Triennial Prize. This was provided for in the will of the late J. Mason Warren, in honor of the memory of his father, John C. Warren. The trustees of the fund are those of the Massachusetts General Hospital, and a committee of the staff of the hospital constitutes the judges. The prize was won for the first time in 1871, by Dr. H. C. Wood, of Philadelphia. No essays were presented for the prize of 1874, for which year the subject proposed was "The Elimination of Drugs by the Mammary Gland." No special subject is given for 1877, but competitors have the full latitude allowed by the will to choose any subject in physiology, surgery, or pathological anatomy involving original researches. The sum offered is a handsome one, and we hope will call out many papers. A prize of this nature may, like the quality of mercy, be called twice blest, as the honor which falls upon the successful competitor is reflected on the memory of the eminent surgeon in whose commemoration the prize was established.—*Boston Medical and Surgical Journal.*

